# Polycyclic Aromatic Hydrocarbons as Plausible Prebiotic Membrane Components

**DOI:** 10.1007/s11084-012-9292-3

**Published:** 2012-07-15

**Authors:** Joost Groen, David W. Deamer, Alexander Kros, Pascale Ehrenfreund

**Affiliations:** 1 Leiden Institute of Chemistry, Leiden University, 2333 CC Leiden, The Netherlands; 2 Department of Chemistry and Biochemistry, University of California Santa Cruz, Santa Cruz, CA 95060 USA

**Keywords:** Primitive membranes, Polycyclic aromatic hydrocarbons, Membrane permeability

## Abstract

Aromatic molecules delivered to the young Earth during the heavy bombardment phase in the early history of our solar system were likely to be among the most abundant and stable organic compounds available. The Aromatic World hypothesis suggests that aromatic molecules might function as container elements, energy transduction elements and templating genetic components for early life forms. To investigate the possible role of aromatic molecules as container elements, we incorporated different polycyclic aromatic hydrocarbons (PAH) in the membranes of fatty acid vesicles. The goal was to determine whether PAH could function as a stabilizing agent, similar to the role that cholesterol plays in membranes today. We studied vesicle size distribution, critical vesicle concentration and permeability of the bilayers using C_6_-C_10_ fatty acids mixed with amphiphilic PAH derivatives such as 1-hydroxypyrene, 9-anthracene carboxylic acid and 1,4 chrysene quinone. Dynamic Light Scattering (DLS) spectroscopy was used to measure the size distribution of vesicles and incorporation of PAH species was established by phase-contrast and epifluorescence microscopy. We employed conductimetric titration to determine the minimal concentration at which fatty acids could form stable vesicles in the presence of PAHs. We found that oxidized PAH derivatives can be incorporated into decanoic acid (DA) vesicle bilayers in mole ratios up to 1:10 (PAH:DA). Vesicle size distribution and critical vesicle concentration were largely unaffected by PAH incorporation, but 1-hydroxypyrene and 9-anthracene carboxylic acid lowered the permeability of fatty acid bilayers to small solutes up to 4-fold. These data represent the first indication of a cholesterol-like stabilizing effect of oxidized PAH derivatives in a simulated prebiotic membrane.

## Introduction

A common feature of all cellular life is the presence of boundaries composed of amphiphilic molecules that self-assemble as bilayers. These cell membranes are composed of phospholipids mixed with polycyclic compounds such as cholesterol, but it is likely that the first membranes consisted of much simpler amphiphilic species. Potential sources of these amphiphiles include synthesis through Fischer-Tropsch reactions associated with volcanism (McCollom and Seewald 2007; Rushdi and Simoneit 2001; Simoneit 2004) as well as extraterrestrial delivery of organic compounds during the early history of the solar system and the young Earth. For instance, Chyba and Sagan (1992) estimated the extraterrestrial delivery of carbon to be in the order of 10^9^ kg per year during the early heavy bombardment phase. Carbonaceous meteorites contain pristine organic compounds, among them are monocarboxylic acids (Sephton 2002). These range from C_2_ (acetic acid) to C_12_ (dodecanoic acid), with decreasing abundance as the carbon number increases. A suite of compounds extracted from the Murchison meteorite by organic solvents are amphiphilic and assemble into membranous vesicles (Deamer 1985; Deamer and Pashley 1989).

From these and other studies, it seems likely that monocarboxylic acids (i.e. fatty acids) with chain lengths ranging between 8 and 12 carbons were able to be constituents of primitive cell membranes on the early Earth. In support of this hypothesis it was previously shown that pure fatty acids are able to self-assemble into vesicles in aqueous dispersions when the pH is similar to the pK_a,_ because deprotonated and protonated head groups form hydrogen bonds that stablize bilayer structures (Monnard and Deamer 2002, 2003). Vesicles composed of fatty acid are dynamic assemblies: molecules constantly flip-flop between the inner and outer leaflets and rapidly exchange between the bilayer and the surrounding medium. Fatty acid vesicles can also grow and divide under simulated prebiotic conditions (Zhu and Szostak 2009).

The shortest fatty acid able to form stable vesicles is octanoic acid (Monnard and Deamer, 2003) but only at relatively high concentrations exceeding 100 mM. As carbon number increases the Critical Vesicle Concentration (CVC), defined as the minimal concentration of amphiphiles that allows vesicle formation, decreases. Decanoic acid (DA) is useful as a model system for prebiotic membranes because its CVC is 30 mM at room temperature. A recent study showed that a mix of C_6_-C_9_ fatty acids added to decanoic acid lowers the CVC significantly (Cape et al. 2011).

Pure fatty acid vesicles are relatively permeable to ionic and polar solutes. For instance, decanoic acid vesicles cannot capture dyes or tRNA (Maurer et al. 2009), which means these membranes would need to incorporate stabilizing compounds if they were to serve as containers for important molecules such as RNA in primitive forms of cellular life. A few prebiotically plausible stabilizers have been discovered that lower the CVC, reduce membrane permeability and provide stabilization over alkaline pH ranges. These include fatty alcohols and monoacyl glycerol derivatives (Monnard and Deamer 2003; Maurer et al. 2009) or mixed cationic and anionic amphiphiles (Namani and Deamer 2008).

Another source of potential membrane stabilizing compounds are polycyclic aromatic hydrocarbons (PAHs) which are abundant in the ISM (Gredel et al. 2010) galactic and extragalactic regions, protoplanetary disks and solar system objects (Tielens 2008; Peeters et al. 2011). These accumulate into planetesimals from which solar system bodies, such as planets, comets and asteroids form. Carbonaceous meteorites are fragments of asteroids and comets and contain ~3 % organic matter. Polycyclic aromatic hydrocarbons such as pyrene and fluoranthene, oxidized aromatic species ( 9-fluorenone, 9-anthrone, 9,10-anthraquinone, and phenanthrenedione) have been identified in the soluble phase and substantial amounts of kerogen-type material composed largely of polymerized aromatics are present in the insoluble phase (Ashbourn et al. 2007). The Aromatic World hypothesis (Ehrenfreund et al. 2006) postulates that aromatic material, being more resistant to degradation by radiation and higher temperatures, may have had functional and structural roles in the emerging of early life forms. Although macromolecular carbon consisting of aromatic units is often perceived as inert, decomposition of these networks by hydropyrolysis can release smaller PAH molecules (Mautner et al. 1995). Oxidized PAHs would then be available for further reactions, thereby adding more diversity to the carbon inventory (Cody and Alexande 2005).

PAHs have the potential to fulfill a variety of functions in prebiotic container chemistry. For instance, amphiphilic PAHs could increase resistance of vesicles to divalent cations, which at relatively low concentrations cause collapse of fatty acid vesicles (Monnard et al. 2002). They could also increase the range of salinity, temperature and pH at which vesicles are stable. Another function of amphiphilic PAH derivatives might be to decrease the permeability of the membranes so that they can entrap RNA in a primitive cell yet remain permeable to smaller nutrient solutes.

Cholesterol and other sterols in contemporary eukaryotic cell membranes serve to reduce permeability and stabilize phospholipid bilayers over a range of environmental conditions (Raffy and Teissie 1999). In prokaryotes, hopane derivatives called hopanoids, detected in 2.7 Gy old Archean shales (Brocks et al. 1999), seem to fulfill a similar role by e.g. reducing membrane permeability (Welander et al. 2009). In the research reported here, we studied whether PAHs can function as plausible prebiotic analogues of these polycyclic molecules by incorporating different polycyclic aromatic hydrocarbon species in fatty acid vesicles.

## Materials and Methods

Decanoic acid, nonanoic acid, octanoic acid, heptanoic acid, hexanoic acid, 1-decanol, pyrene, 1-hydroxypyrene, 9-anthracene carboxylic acid, 1-pyrene carboxaldehyde, 9-fluorenone, 1,4 chrysene quinone and 1 M Tris buffered solution (pH 7.5) were obtained from Sigma Aldrich. All chemicals were of the highest available purity grade.

Vesicle solutions contained 60 mM of PAH/decanoic acid (in a 1:10 ratio unless stated otherwise) and a fatty acid mix (FA mix) of 80 mM of C_6_-C_9_ fatty acids (20 mM each). For convenience the mixtures will be expressed by their PAH/decanoic acid ratio, but the FA mix is always included because a mixture of fatty acids is both prebiotically more plausible (Sephton 2002) and because it stabilizes the vesicles (Cape et al. 2011).

To prepare fatty acid vesicles, a dried film of fatty acid (C_6_-C_10_) and PAH was dispersed in 10 mM Tris buffer at 43 °C. This temperature was used to keep decanoic acid well above its melting point of 32 °C (Monnard and Deamer 2003). The vesicle suspensions (10 ml) were titrated to pH 7.4 using 1 M NaOH and left at room temperature to equilibrate overnight. Solutions without PAH derivatives were prepared as above using 60 mM decanoic acid and the fatty acid mix.

Incorporation of different PAH species in the fatty acid bilayer was determined by epifluorescence microscopy as PAHs are fluorescent with excitation wavelengths in the UV-range. Phase-contrast and epifluorescence microscopy was carried out with a Zeiss Axiovert 200 inverted microscope. The illumination source was a HBO 103 W/2 mercury pressure short-arc lamp with an ultraviolet filter set (excitation filter of 365 nm) for epifluorescence microscopy and a HAL 100 halogen lamp for phase-contrast microscopy. All images were taken at room temperature. Photoshop CS4 (Adobe) was used to adjust brightness and contrast to optimize images. Dynamic Light Scattering was performed with a Malvern Zetasizer Nano ZS using the size measurement function and a scattering angle of 173°. Optimal measurement position and attenuator settings were chosen automatically.

The CVC of fatty acids has been measured previously by UV–vis spectrometry (Apel et al. 2002), static light scattering (Chen and Szostak, 2004) and merocyanine-540 assays (Dixit and Mackay 1983). As an alternative, we used conductimetric titration (Briz and Velásquez 2002) to determine the CVC of fatty acid vesicles. When the conductance of a micellar solution of a fatty acid is measured as fatty acid concentration increases, all of the fatty acid anions and counterions are available to carry ionic current. However, when the concentration of fatty acid exceeds the CVC, half of the fatty acid anions and counterions become unavailable because they are incorporated in the inner leaflet of the vesicle bilayer membranes. When concentration is plotted against conductivity the slope decreases above the CVC, and the intersect of the two linear fits gives the value of CVC (Williams et al. 1955).

The CVC was determined by conductimetric titration. The sample temperature was kept at 25.0 ± 0.1 °C with a thermal circulating water bath. An analogue electrical conductivity meter and an electrode with cell constant of 0.55 cm^−1^ were used to measure electrical conductivity. The cell constant was determined by calibration with KCl samples of known concentration. Titration was performed by successive dilution of the sample with 10 mM Tris buffer (pH 7.4), lowering the decanoic acid concentration of the sample 3 mM at a time. Solutions were allowed to equilibrate a few minutes after dilution until a stable conductivity measurement was obtained. CVC values were calculated using the Williams method. The linear fits of data points at high concentration (above CVC) and low concentration (below CVC) had an R^2^ > 0.99.

The permeability of the mixed membranes to small solutes was studied using a turbidity assay (Monnard and Deamer 2003; Cohen and Bangham 1972). When solutes are added to vesicles in solution, osmotic pressure causes the vesicles to shrink, resulting in increased light scattering (measured as absorbance). If the membranes are permeable, solute molecules diffuse through the membrane and the vesicles swell, lowering the absorbance. The initial rate at which absorbance decreases is a measure of the relative permeability of the membrane to that solute (Apel et al. 2002).

Permeability measurements were performed according to Apel et al. (2002). An aliquot of each vesicle preparation (0.9 ml) was added to a 1 ml quartz cuvette. Absorbance was measured at 600 nm with a VarianCary50 UV/Vis Spectrophotometer. After 20 s, 100 μl solute was added and mixed thoroughly for a final 0.1 M solute concentration. Measurements were performed every 10 s, and data points were fitted to exponential decay using Origin Pro 8.0. The initial rate of permeation in Abs/s was determined by extrapolating to zero (point of solute injection) and calculating the first derivative. Fitting the curve to an exponential decay function provided a mean lifetime used to calculate permeability coefficients. We measured the permeability of different PAH/fatty acid membranes to KCl and sucrose. For each PAH species three samples were prepared and each sample was measured three times.

## Results

### Microscopy and DLS of Vesicle Solutions

**Fig. 1:**
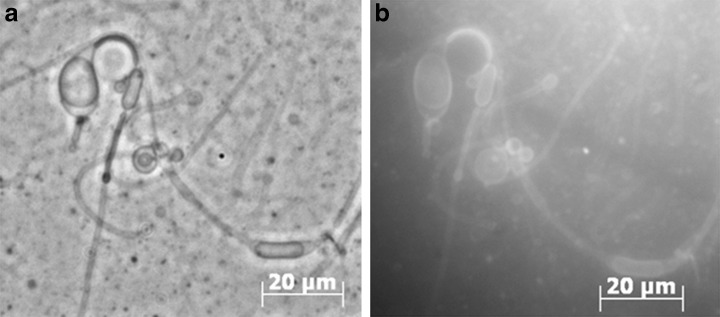
Phase-contrast **a** and fluorescence **b** microscopy of 0.3 mM pyrene + 59.7 mM DA (200:1) + FA mix. Tubular structures are formed by vesicles adhering to the coverslip or glass slide. Pyrene fluorescence is clearly localized to the membrane

Phase-contrast and fluorescence microscopy of vesicle preparations with a 1:200 ratio of pyrene/decanoic acid are shown in Fig. 1. PAHs are fluorescent under UV light and incorporation can therefore be determined by fluorescence microscopy. The vesicles were heterogeneous, ranging from 100 nm to 5 μm with a mean of 200 nm. Vesicles were largely spherical at first, but tubular vesicles dominated a few minutes later after attaching to the surface of the glass slide or coverslip (Apel et al., 2002). Incorporation of PAHs did not influence mean vesicle sizes or the size distribution. Vesicles of pure decanoic acid disappeared at pH 7.6, but incorporation of 1-hydroxypyrene had a modest stabilizing effect, with vesicles still apparent at pH 8.1.

### PAH Incorporation

**Fig. 2:**
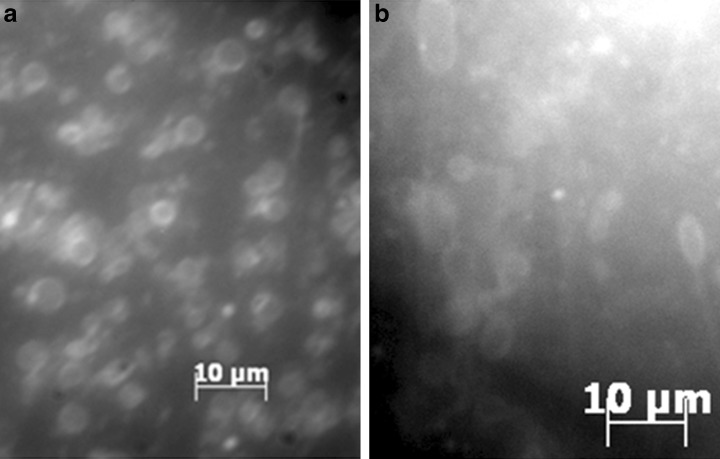
Fluorescence microscopy of **a** 5.5 mM 1-hydroxypyrene + 54.5 mM DA (1:10) + FA mix and **b** 5.5 mM 9-anthracene carboxylic acid + 54.5 mM DA (1:10) + FA mix samples. Fluorescence is clearly localized to the membrane boundary

UV Fluorescence microscopy showed that PAH derivatives could be incorporated into the membrane in different concentrations. Pyrene could be incorporated in a 1:200 mole ratio with decanoic acid while 1-hydroxypyrene (Fig. 2-a) and 9-anthracene carboxylic acid (Fig. 2-b) were incorporated up to 1:10 ratios. Only 1:50 ratios of 9-fluorenone and 1-pyrene carboxaldehyde could be incorporated before macroscopic aggregates formed or PAHs precipitated. In some cases (1-pyrene carboxaldehyde, 9-fluorenone), 10 freeze-thaw cycles using liquid nitrogen to homogenize the bilayers prevented the formation of macroscopic aggregates.

### CVC Measurements

**Fig. 3:**
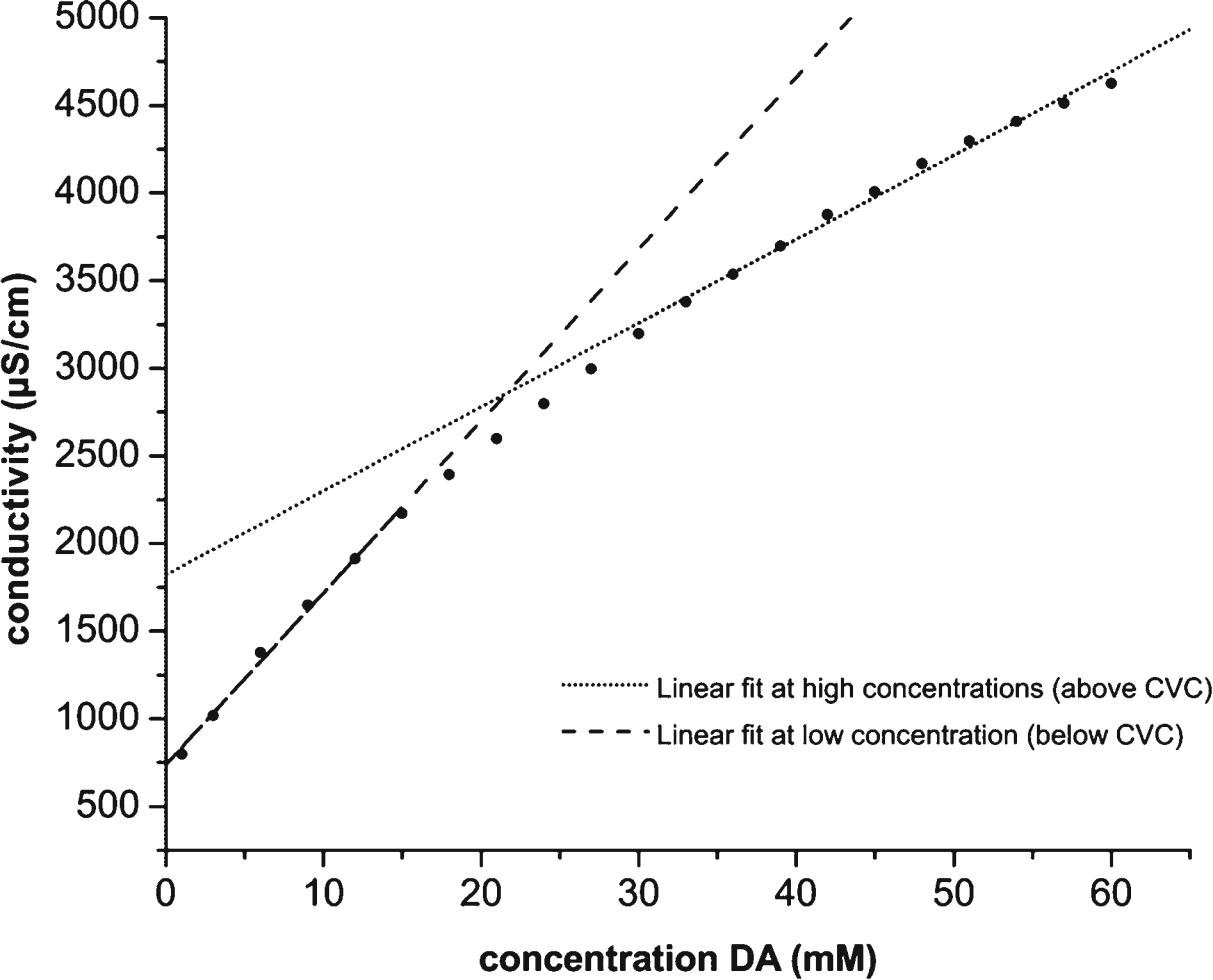
Conductimetric titration of a 5.5 mM 1-hydroxypyrene + 54.5 mM DA (1:10) + FA mix sample. The measured CVC is 21.6 mM and this coincides with the formation of macroscopic fluffy aggregates

**Fig. 4:**
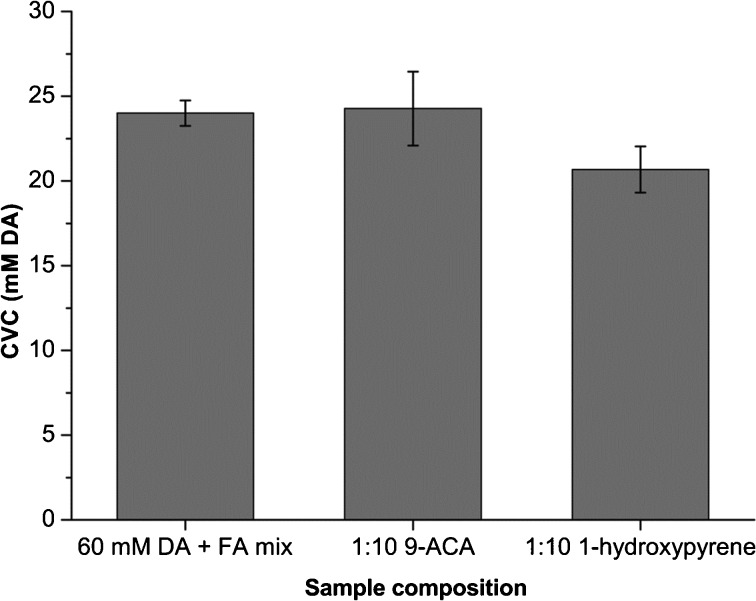
CVC values determined by conductimetric titration. CVC’s are: 24.00 ± 0.7 mM for 60 mM DA + FA mix samples, 24.3 ± 2.2 mM for 1:10 9-anthracene carboxylic acid samples and 20.7 ± 1.4 mM for 1:10 1-hydroxypyrene samples. Error bars represent standard deviations

Conductimetric titration was performed on vesicle preparations to determine CVC values. Figure 3 shows CVC measurements for a 1:10 1-hydroxypyrene / decanoic acid sample, the measured CVC values (Fig. 4) are in the range of previously published values (Monnard and Deamer 2003; Cape et al. 2011). Of the PAH derivatives that were tested, only 1-hydroxypyrene showed a significant reduction in CVC, forming fluffy macroscopic aggregates around the measured CVC value. All other samples became completely clear when diluted below the measured CVC values.

### Permeability Assays

**Fig. 5:**
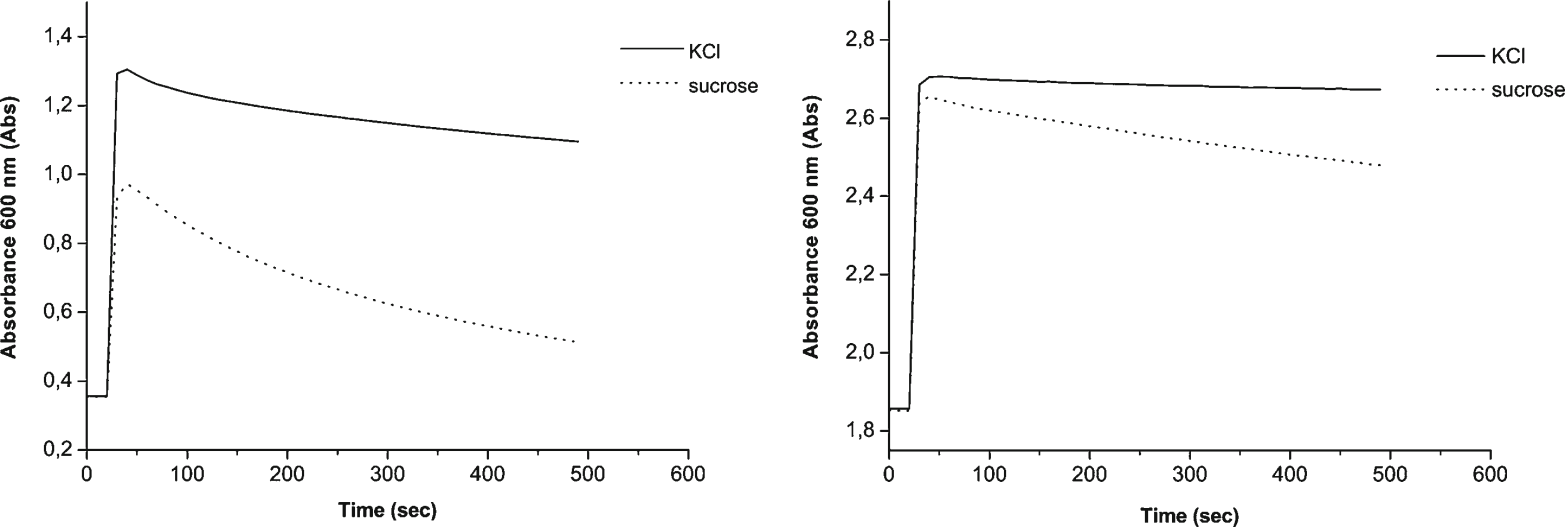
Permeability assays of a 60 mM DA + FA mix sample (*top*) and a 1:10 1-hydroxypyrene sample (*bottom*). Injection of 0.1 M of solute at t = 20 s. The absorbance decrease due to swelling of vesicles by solute passing the membrane is significantly slower in the 1-hydroxypyrene samples

**Fig. 6:**
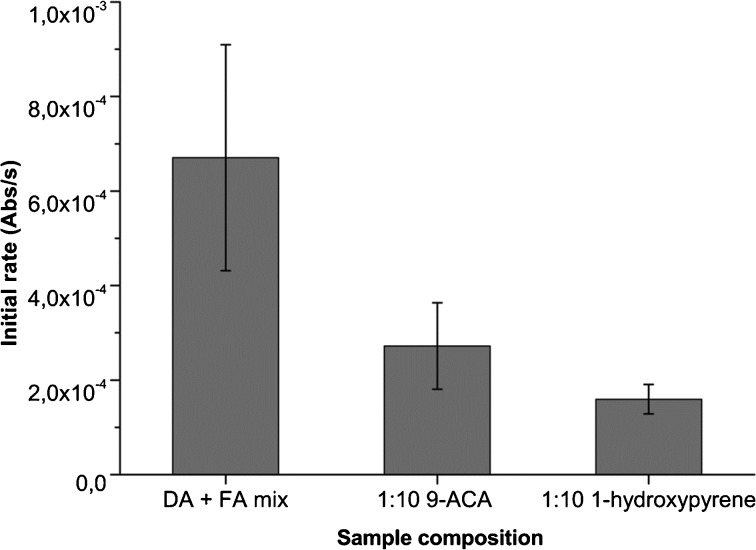
Initial rates of absorbance loss due to reswelling of vesicles by KCl permeation of a 60 mM DA + FA mix, 1:10 9-ACA + FA mix and a 1:10 1-hydroxypyrene + FA mix sample. Values are calculated by fitting data to exponential decay and extrapolating to t = 20 s (time of solute injection). Error bars represent standard deviations

The influence of PAHs on the permeability of fatty acid bilayers to sucrose and KCl was measured using UV–vis spectrophotometry. Initial rates were determined by extrapolating to zero (time of solute injection) and determining the slope of the curve. The data matched an exponential decay curve with R^2^ ≥ 0.995. Figure 5 shows permeability assays for a pure fatty acid sample and a sample with 1:10 1-hydroxypyrene. Permeability of the membranes to both KCl and sucrose was significantly decreased by incorporation of 1-hydroxypyrene. The initial rates for permeability to KCl of different PAH incorporations are shown in Fig. 6 (values are based on ≥ 3 samples).

**Fig. 7:**
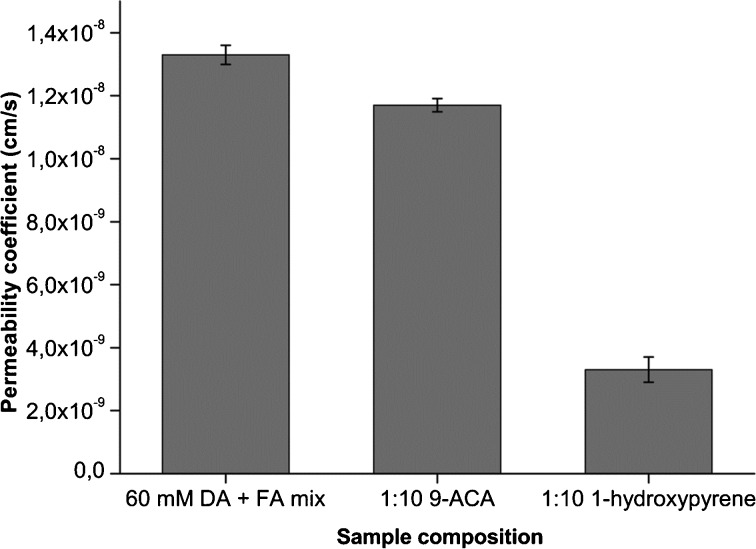
Permeability coefficients of sucrose calculated by determination of the decay constant by fitting the data to exponential decay. The permeability coefficient is lowered ~4 fold by 1-hydroxypyrene incorporation. Error bars represent standard deviations

Both 1-hydroxypyrene and 9-anthracene carboxylic acid significantly decreased the permeability of fatty acid membranes to KCl by 4.2- and 2.5-fold, respectively. Permeability coefficients for sucrose were determined according to Chakrabarti & Deamer (1992). The interior solute concentration of vesicles obeys A(t)_int_ = A(eq)_int_ (1-e^λt^), where A(t)_int_ is the interior concentration of solute at time t, A(eq)_int_ is the interior solute concentration at t = infinity and λ is the decay rate. Since 0.1 M of solute is added and the osmotic gradient should disappear at A_int_ = A_ex_, A(eq)_int_ can be assumed to be 0.1 M (the total interior volume of the vesicles is negligible compared to the volume of bulk medium), so A(t)_int_ = 0.1–0.1*e^-t/τ^. The mean lifetime (τ) can be obtained directly from fitting the data to exponential decay, and permeability coefficients can be obtained by *P* = (r/3) λ. Figure 7 shows the measured coefficients.

## Discussion

PAHs are present in many space environments and likely contributed to the carbon inventory on the early Earth delivered during the heavy bombardment phase through impacts of small solar system bodies (Chyba and Sagan 1992; Gomes et al. 2005), as well as abiotic synthesis on the early Earth. These molecules are very resistant to radiation and degradation at higher temperatures and can potentially be oxidized to form more soluble compounds that might have played a role in early membrane chemistry (Ehrenfreund et al. 2006). The identification of prebiotically plausible molecules that can influence the physical and chemical characteristics of fatty acid vesicles is essential for understanding membrane chemistry of the early Earth. A recent study (Cape et al. 2011) confirmed the ability of naptho[2,3a]pyrene and perylene to photochemically induce trans-membrane charge transport thereby acting as a primitive pigment system (Deamer 1992). However, these hydrophobic PAHs could not be incorporated in high concentrations in fatty acid bilayers and had no measurable effect on membrane stability.

In the study reported here, we investigated the possibility that oxidized PAH derivatives can act as membrane stabilizers by reducing CVC or membrane permeability to small solutes. We successfully incorporated several oxidized PAH derivatives in fatty acid membranes as confirmed by phase-contrast and epifluorescence microscopy. Both 1-hydroxypyrene and 9-anthracene carboxylic acid could be incorporated in up to 1:10 PAH/DA ratios while 1-pyrene carboxaldehyde, 9-fluorenone, 1,4-chrysene quinone and pyrene could be incorporated in lower ratios (see Table 1). Size distribution was determined by DLS (data not shown) and showed a very heterogeneous population of vesicles ranging in diameter from 100 nm to 5 μm with a mean diameter of approximately 200 nm. PAH incorporation had no measurable effect on vesicle size or morphology.

**Table 1:** List of performed experiments

Sample	Maximum solubility ratio (PAH/DA)	mM DA at CVC	Incorporation confirmed by fluorescence microscopy	Permeability assay performed
decanoic acid	x	30.5 ± 2.5	x	x
decanoic acid + fatty acid mix	x	24.0 ± 0.75	x	v
DA + 1-decanol	1:10^a^	18.9	x	x
DA + 1,4 chrysene quinone	1:200	33	yes	x
DA + pyrene	1:200		yes	x
DA + 9-fluorenone	1:100	32.0	no^b^	x
DA + 1-PCA	1:200	30.7	yes	x
DA + 1-hydroxypyrene + FA mix	1:10	20.7 ± 1.4	yes	v
DA + 1-PCA + FA mix	1:50 (10x freeze-thaw)	23.7 ± 0.5	yes	v
DA + 9-fluorenone + FA mix	1:20	25.0 ± 1.1	no^b^	x
DA + 9-ACA + FA mix	1:10	24.3 ± 2.2	yes	v

All mixed membranes tested. Addition of C_6_-C_9_ fatty acids lowers CVC (Cape et al. 2011). 9-fluorenone incorporation cannot be visualized by epifluorescence microscopy due to quenching (Biczók et al. 1997)

^a^(Monnard & Deamer 2003)

^b^(Biczók et al., 1997)

Incorporation of 1-hydroxypyrene allowed vesicles to be stable at pH 8.1, while pure fatty acid samples only formed micelles. The stabilization of vesicle suspensions at alkaline pH due to hydrogen bonding of decanoate with a hydroxyl group was previously established for decanol and glycerol monodecanoate (Monnard and Deamer 2003; Maurer et al. 2009).

Measurements of CVC values by conductimetric titration produced reproducible values that coincided with the concentrations at which vesicle solutions become completely transparent. Dissociative ionization upon dilution might produce small errors in the values, but the measured CVC’s (~20–25 mM), are comparable to previously reported values (Monnard and Deamer 2003; Cape et al. 2011). Incorporation of oxidized PAH derivatives did not affect CVC values, the only exception being 1-hydroxypyrene which produced a statistically significant CVC reduction. The formation of fluffy aggregates in 1-hydroxypyrene samples around the CVC requires further investigation. One possibility is that upon dilution the fatty acid bilayers reach a critical 1-hydroxypyrene concentration at which point vesicles aggregate.

The high permeability of fatty acid vesicles has certain advantages in a prebiotic setting because small molecules would be able to cross a membrane barrier without requiring the highly evolved protein transport system used by life today. However, high permeability also means that fatty acid vesicles are unable to encapsulate large molecules such as dyes and tRNA (Maurer et al. 2009). A balance is needed in which smaller nutrient molecules can be transported into a primitive cell while larger molecules that perform essential functions such as catalysis can be maintained in the vesicle lumen.

Our measurements of the permeability of mixed membranes for small solutes produced the following significant results. Incorporation of 1:10 1-hydroxypyrene/DA lowered the initial rate of permeation of KCl 4.2 fold while 1:10 9-anthracene carboxylic acid/DA lowered the permeation of KCl 2.5 fold. The decrease in membrane permeability to KCl by incorporation of 1-hydroxypyrene and 9-anthracene carboxylic acid is in the same order of magnitude in which cholesterol decreases K^+^ and Na^+^ leakage in modern phospholipid membranes, which is 3-fold (Haines 2001). The influence of hopanoids on the permeability of prokaryotic membranes is still relatively unexplored.

The permeability coefficient of sucrose was lowered 4-fold by 1-hydroxypyrene incorporation, from 1.3 × 10^−8^ cm/s to 3.3 × 10^−9^ cm/s. Comparing this to longer chain amphiphiles, the permeability coefficients of oleate vesicles to monosaccharides like ribose are in the ~10^−8^ range (Mansy et al. 2008) while the permeability coefficient of phosphatidylcholine membranes to sucrose is 2.1 × 10^−13^ cm/s (Brunner et al. 1980). While 1-hydroxypyrene provides a significant lowering of the membrane permeability to KCl and sucrose, small molecules like glycerol can still pass these membranes very rapidly (data not shown).

In summary, the permeability of decanoic acid membranes for small solutes is significantly reduced by 1-hydroxypyrene, although the permeability is larger compared to current day membranes composed of longer chain phospholipids. These data represent the first indication of a cholesterol-like stabilizing effect of oxidized PAH derivatives in a simulated prebiotic membrane.

## Abbreviations

1-PCA: 1-pyrene carboxaldehyde
9-ACA: 9-anthracene carboxylic acid
CVC: critical vesicle concentration
DA: decanoic acid
DLS: Dynamic Light Scattering
FA mix: fatty acid mix: 80 mM of C_6_-C_9_ fatty acids (20 mM each)
PAH: polycyclic aromatic hydrocarbons
